# Exploring the Genetic Characteristics of Two Recombinant Inbred Line Populations via High-Density SNP Markers in Maize

**DOI:** 10.1371/journal.pone.0052777

**Published:** 2012-12-27

**Authors:** Qingchun Pan, Farhan Ali, Xiaohong Yang, Jiansheng Li, Jianbing Yan

**Affiliations:** 1 National Maize Improvement Center of China, China Agricultural University, Beijing, China; 2 National Key Laboratory of Crop Genetic Improvement, Huazhong Agricultural University, Wuhan, China; 3 Cereal Crops Research Institute (CCRI) Nowshera, Kyber Pukhtunkhwa, Pakistan; China Agricultural University, China

## Abstract

Understanding genetic characteristics can reveal the genetic diversity in maize and be used to explore evolutionary mechanisms and gene cloning. A high-density linkage map was constructed to determine recombination rates (RRs), segregation distortion regions (SDRs), and recombinant blocks (RBs) in two recombinant inbred line populations (RILs) (B73/By804 and Zong3/87-1) generated by the single seed descent method. Population B73/By804 containing 174 lines were genotyped with 198 simple sequence repeats (SSRs) markers while population Zong3/87-1 comprised of 175 lines, were genotyped with 210 SSR markers along with 1536 single nucleotide polymorphism (SNP) markers for each population, spanning 1526.7 cM and 1996.2 cM in the B73/By804 and Zong3/87-1 populations, respectively. The total variance of the RR in the whole genome was nearly 100 fold, and the maximum average was 10.43–11.50 cM/Mb while the minimum was 0.08–0.10 cM/Mb in the two populations. The average number of RB was 44 and 37 in the Zong3/87-1 and B73/By804 populations, respectively, whereas 28 SDRs were observed in both populations. We investigated 11 traits in Zong3/87-1 and 10 traits in B73/By804. Quantitative trait locus (QTLs) mapping of SNP+SSR with SNP and SSR marker sets were compared to showed the impact of different density markers on QTL mapping and resolution. The confidence interval of QTL Pa19 (*FatB* gene controlling palmitic acid content) was reduced from 3.5 Mb to 1.72 Mb, and the QTL Oil6 (*DGAT1-2* gene controlling oil concentration) was significantly reduced from 10.8 Mb to 1.62 Mb. Thus, the use of high-density markers considerably improved QTL mapping resolution. The genetic information resulting from this study will support forthcoming efforts to understand recombination events, SDRs, and variations among different germplasm. Furthermore, this study will facilitate gene cloning and understanding of the fundamental sources of total variation and RR in maize, which is the most widely cultivated cereal crop.

## Introduction

Maize (Zea mays L.) is an important crop used for food and feed while its production and consumption are the highest among cereal crops [1]. It has recently gained additional interest as a renewable energy plant due to its high biomass potential. To be grown magnificently under different climatic conditions across the globe, breeders maintain maize at high genetic diversity. Maize is a model crop for genetic investigations because of its high degree of genetic diversity [2] and ability of scientists to manipulate its genomic sequence via insertions, deletions, or recombination events [3]. High-density single nucleotide polymorphism (SNP) markers are widely used for such investigations and are an effective tool for determining the genomic and genetic characteristics of maize [4]. Compared with traditional markers such as random amplified polymorphic DNAs (RAPDs), simple sequence repeats (SSRs), and restriction fragment length polymorphisms (RFLPs), SNPs are of higher density, cost effective, and yields more genetic information. To date, tens of millions of SNPs [5] and more than 1600 SSR markers (www.maizegdb.org) have been identified in various maize lines. Moreover, the investigative utility of this large number of maize SNPs is enhanced because the cost of genotyping is decreasing steadily [6].

Maize lines have significant genetic diversity and phenotypic variation, with DNA varying significantly even between two inbred lines [7]. High-density markers provide a promising tool to identify recombinant blocks (RBs), recombination rates (RRs) and segregation distortion regions (SDRs) in maize genome by constructing ultra-high density map. Crossover sites do not occur randomly on chromosomes, indeed recombination hot spots correlate positively with gene density, and recombination cold sites occur in centromeres and intergenic regions [4], [8]. Several studies have used quantitative trait locus (QTL) mapping of RBs in segregating populations of *Arabidopsis thaliana* and maize but reliable results are hard to be obtained because the coverage of genome by limited numbers of markers [9]–[10]. Furthermore, to procure a precise degree of how recombination rates differ across the genome has implications for understanding the molecular basis of recombination, its evolutionary importance and the distribution of linkage disequilibrium in natural populations [11].

Segregation distortion is a complex genetic mechanism involved in recombination and evolutionary change [12]–[13]. SDRs in many plants do not follow the distribution according to Mendel law of segregation [14]–[16]. Comparing and integrating SDRs in different populations is extremely useful when analyzing the genetic basis of segregation and understanding different genetic characteristics [17].

Development of segregating populations is extremely important for the use of QTL mapping to identify genes of interest. It has been difficult to obtain accurate recombination information with a limited number of markers and small population sizes [18], so the resulting QTL confidence interval (CI) is usually large, i.e., 10–30 cM. However, new high-throughput and low-cost DNA genotyping technologies now provide informative genome-wide and high-density markers for mapping. High-density markers can significantly improve the resolution of QTL mapping, facilitating the discovery of additional recombination events and exact recombination breakpoints. For example, using high-density maps of two different recombinant inbred lines (RIL) rice populations genotyped by sequencing identified two QTLs in regions less than 200-kb containing the *GS3*, *GW5*/*qSW5* genes controlling grain length and width, respectively [19]–[20].

In this study, oligo pool assay (OPA) 1536 SNP markers and about 200 SSR markers were used to genotype two RIL maize populations, yielding comprehensive information about RB, RR, and SDR variations at the whole-genome level. We also re-mapped QTLs affecting 21 different traits and compared the power and resolution of QTL mapping using different density markers, providing basic information for use by the breeding community to increase maize quality and production.

## Materials and Methods

### The two RIL populations

Two RIL populations were used in this study: B73/BY804 and Zong3/87-1 [21]–[22]. The Population B73/BY804 comprised of 174 lines and population Zong3/87-1 comprised of 175 lines. The RILs and their parents were genotyped using Golden Gate assays (Illumina, San Diego, CA, USA) containing 1536 SNPs [23]. The population B73/BY804 was genotyped with 198 SSR markers [22] and Zong3/87-1 was genotyped with 210 SSR markers [21]. Among the 1536 SNPs, 1467 have precise physical positions on different chromosomes, and the remaining reads were either unique or nonaligned to the reference genome B73 (http://www.panzea.org).

### Identification of outliers within the RILs

Maize is a cross-pollinated crop, highly prone to contamination when constructing pure inbred or RIL populations. Several generations are involved in creating RILs, and contamination can occur in every generation. Here, four types of contamination (A to D) were defined: A: pollen contamination within the population prior to the S3 generation; B: pollen contamination within the population after the S3 generation; C: contamination by outer group or foreign pollen from different lines in early generations (S3 or before); and D: contamination by foreign pollen from different lines after the S3 generation. The availability of high-density markers enabled the detection of all the above-mentioned contamination types when generating RILs for genetic studies. Several generations of selfing from F1 to F6 ensured very low rates of heterozygosity in the lines and near kinship to the parental lines for accurate mapping and high-quality genetic characterizations.

Using these properties, a specific method for identifying lines was used in which the level of heterozygosity was determined for all the RILs and parents. The levels of nonpolymorphism, polymorphism, and heterozygosis were determined for 1467 SNP markers, which harbored precise genomic information for B73/By804 and Zong3/87-1 RILs and the four parents used in this experiment. The rate of heterozygosity was calculated using the following three formulas:
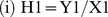
Where, H1 is the rate of heterozygosity, Y1 is the number of heterozygotic site markers, and X1 is the total number of used SNP markers;
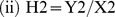
Where, H2 is the rate of heterozygosity in nonpolymorphic sites (nonpolymorphic markers in parents), Y2 is the number of heterozygotic site markers in nonpolymorphic sites (nonpolymorphic markers in parents), and X2 is the total number nonpolymorphic markers;
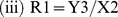
Where, R1 is the ratio of mixed sites (such as the grey color of the genome in Figure S1), and Y3 is the number of polymorphic sites in a line of nonpolymorphic sites of the populations (nonpolymorphic markers in parents but polymorphic sites in a RIL). By calculating the proportion of heterozygosity and familial relationship, different lines were identified, and the lines creating any ambiguity during the assessment of genetic architecture were excluded from the analysis.

### Linkage map construction

The following steps were used to construct the genetic linkage map. SNP marker information was obtained from http://www.panzea.org, and SSR marker information was obtained from http://www.maizeGDB.org and http://www.maizesequence.org. Then, the markers with polymorphic and location information were used to construct the genetic map. The forward primer position of a SSR was used as the marker position. Finally, these markers were arranged according to their physical location on the maize AGP v2 reference sequence (http://www.maizeGDB.org) and the markers having linkage distances exceeding 30 cM were deleted. The markers in the double cross region exceeding 5 Mb were considered as missing. R/qtl package command “est.map” was used to construct the linkage map, selecting the kosambi function. The “fill.geno” command and the viterbi method were used to fill the missing genotyping data [24]. Three sets of density markers were used to construct the linkage map: SNP+SSR (combining the SNP and SSR markers), SNP (SNP markers only), and SSR (SSR markers only).

### Recombinant blocks

RBs can be defined as the fragments of chromosomes inherited by the offspring from each parent. The RBs in this experiment were identified for the entire set of chromosomes and are shown as red or blue colored segments (Figure S3). Each red or blue colored fragment from different parents was considered as an individual block. Chromosomal recombination events occur only three to five times on each chromosome on average during the continuous selfing in maize, which was also confirmed by re-sequencing [25]. The number of RB was analyzed using markers information to distinguish both the bin breakpoint from the two parents of each RIL and the total block numbers of all chromosomes for each line of both RILs derived from different parents.

### Recombination rates

Recombination rates was determined by dividing the genetic distance (cM) between two markers by the physical distance (Mb), i.e., cM/Mb. Neighboring markers that were less than 0.2 Mb apart were excluded from the analysis to increase the accuracy and avoid genotyping errors. Hotspots were considered as regions between two markers for which cM/Mb was greater than twice the genome average. When comparing the average recombination rates of the two populations, a 10-Mb window was used to calculate RR.

### Segregation distortion regions

The chi-square test for distortion of markers assessment was used to determine whether the marker ratio for normal Mendelian segregation (1∶1) was significant at the 0.05 level. *X* ^2^ value was calculated using the following formula and the resulting data was used to calculate the P value:
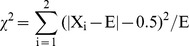
where X_i_ is the number of markers for an individual parent, and E is the expected value of the two parents for each RIL. A marker distortion value was calculated by considering B73 or Zong3 as the male parent and By804 or 87-1 as the female parent. A SDR was defined as a marker having a P value less than 0.05 and the region encompassing the adjacent three markers. The marker segregated distortion (P≤0.05) was defined as the segregated distortion locus (SDLs).

### Phenotyping and QTL mapping

Phenotypic data were collected for 21 traits in the two RIL populations. Ten traits were related to kernel quality in the B73/By804 population: palmitic acid (Pal), stearic acid (Ste), oleic acid (Ole), linoleic acid (Lin), total oil content (Oil), α- tocopherol (AT), δ-tocopherol (DT), γ-tocopherol (GT), total tocopherol (TT), α/γ ratio (A/G). Information about the oil- and tocopherol-related traits has been described [22], [26]. Data on 11 agronomic- and yield-related attributes were collected for the Zong3/87-1 population, including plant height (PH), ear height (EH), ear weight (EW), grain yield (GY), kernels per row (KPR), ear diameter (ED), ear length (EL), row number (RN), length of tassel branch (LTB), 100 kernel weight (100KW), and number of tassel branches (NTB), all of which have been described [21]. The best linear unbiased prediction known as BLUP [27] values of traits phenotyped in multiple environments were used for analysis, and a modified R/qtl package was used for QTL mapping [24]. Mapping method was “*cim*” function, QTL additive and phenotypic variance was estimate from all QTL fitted liner model (R function *lm*). The *lm* function could calculate QTL additive and phenotypic variance. Three sets of markers (SNP+SSR, SNP, and SSR) were used for QTL mapping. A window size of 10 cM at a walking distance of 2 cM was used. The logarithm of odds (LOD) cut-off value was 2.5, and beyond this threshold was defined as a QTL. For each QTL, a 1.0 LOD-drop support interval was defined as the CI.

## Results

### Outlier identification in the two RIL populations

The outliers in the two RIL populations were classified into four distinct groups (A, B, C, and D; Table 1). Theoretically, the RILs were considered as homozygous but we observed some lines having small amount of heterozygosity and few highly heterozygous lines in both the populations. Type B and C mixing were found in B73/By804, whereas type D was found in Zong3/87-1. Type A mixing resulted in homozygous lines in later generations, and in-depth analysis revealed only a few recombination events. It is not clear if the lines had more recombination events because of contamination in early generations or if actual recombination occurred within the lines because of type A contamination. Type B contamination resulted in relatively high heterozygosity ratios in the RILs used in this study. Lines H119, H037, H084, and H075 of B73/By804 populations had 10–20% H1 proportion and non-significant R1 values compared to the group average, indicating that these lines had type B contamination that may have occurred in S3 or S4 generation. Type C contamination was observed and selfing of these lines led to homozygosity but non-parental segments were observed. Four lines in the B73/By804 populations, H101, H147, H004 and H065 had type C contamination. These lines had a normal rate of heterozygosity but had 33.2–34.7% of “new regions”, i.e., mono-polymorphic in parents but polymorphic between RILs and parents (Table 1). Type D lines had a proportion of heterozygosity along with certain non-parental segments on the chromosomes. A single line (R090) from Zong3/87-1 populations had D type mixing, 12% H1 and an 8% R1 ratio. This R1 value is much higher than the group's average, and the line was contaminated in a later generation by pollen from other lines. A total of nine lines were contaminated either by inner-group or outer-group pollen. All these lines were excluded from the subsequent analysis (Table 1).

**Table 1:** Information on heterozygosity in different lines in B73/By804 and Zong3/87-1 populations.

Population	Lines	H1^a^	H2^b^	R1^c^	Type^d^
B73/By804	H037	0.13	0.01	0.002	B
	H075	0.20	0.07	0.003	B
	H084	0.14	0.04	0.003	B
	H119	0.10	0.04	0.002	B
	H004	0.06	0.06	0.337	C
	H065	0.07	0.07	0.332	C
	H101	0.05	0.04	0.347	C
	H147	0.06	0.04	0.337	C
	mean^e^	0.05	0.01	0.01	
	B73	0.07			
	By804	0.03			
Zong3/87-1	R090	0.12	0.08	0.081	D
	mean^f^	0.04	0.02	0.005	
	87-1	0.03			
	Zong3	0.04			

a The heterozygosis rate of a line.

b The rate of heterozygosity in a nonpolymorphic site.

c The ratio of a mixed site.

d Illustrates the types of mixed pollen.

e The mean value in B73/By804 populations.

f The mean value in Zong3/87-1 populations.

### Linkage map construction

Excluding the nine abnormal RILs, 166 and 174 RILs in B73/By804 and Zong3/87-1 populations, respectively, were used to construct the linkage map. In total, 851 polymorphic markers were identified in the B73/By804 population, including 653 SNP markers and 198 SSR markers. The Zong3/87-1 populations had 649 polymorphic, 439 SNP and 210 SSR markers. From these data, we also constructed linkage maps using SNP+SSR, SNP, and SSR markers to compare the effects of different marker density on QTL mapping power and resolution. The total genetic lengths of the maps using three density marker sets (SNP+SSR, SNP, SSR) were respectively 1526.7, 1354.7, and 1336.3 cM in B73/By804, and 1996.2, 1728.8, and 1661.2 cM in Zong3/87-1. The average distance between two markers for the three density maps in the (B73/By804)/(Zong3/87-1) populations was 1.8/3.1 (SNP+SSR), 2.1/4.0 (SNP), and 7.1/8.3 cM (SSR). Comparing the SNP+SSR, SNP with SSR linkage maps had nearly 4-fold and 3-fold improved resolution, respectively, with little changes in the total genetic length (Table S1, S2).

### Segregation distortion

**Figure 1:**
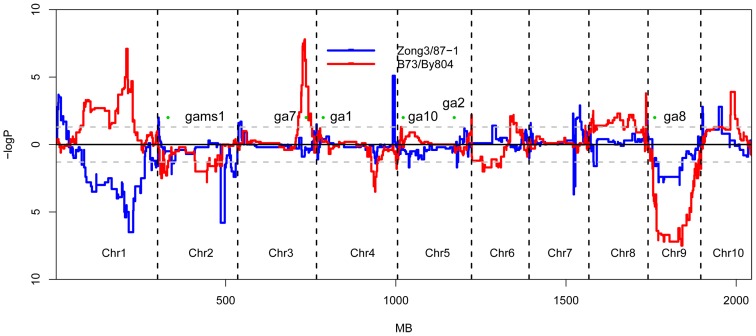
Segregation distortion regions of different chromosomes in two RIL populations. The black horizontal line represents 0 marker distribution, with values above and below this line indicating positive (B73 and Zong3) and negative (By804 and 87-1) segregation distortion, respectively.

The high-density markers were used to determine the most promising mechanism of segregation distortion in these two RILs. B73/By804 and Zong3/87-1 populations had 244 (30%) and 122 (20%) segregation distortion markers, respectively (Table S3). The SDRs varied among chromosomes and were restricted to specific regions within the chromosomes. B73/By804 had 18 SDRs over all the chromosomes, and Zong3/87-1 had 10 SDRs, whereas three regions had the same number of SDRs in both populations. The SDR 7 position coincided with gametophyte factor 7 (*ga7*) on bin 3.09, whereas SDRs 16 and 27 were located on bin 9.02–9.05; Pioneer Composite 1999 maps (http://www.maizegdb.org/) previously showed that *ga8* resides on bin 9.03 (Figure 1; Table S4).

### Recombination rates

**Figure 2:**
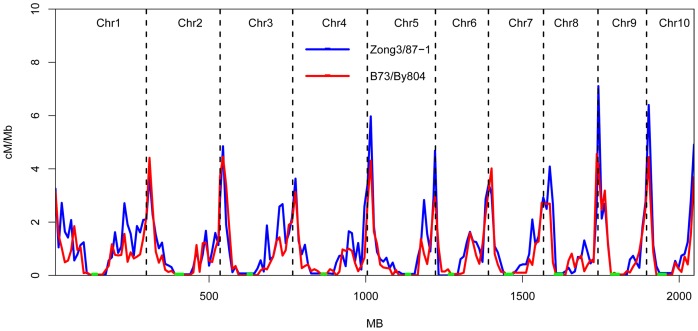
Distribution of recombination rate in two RIL populations. The green bar indicates the centromere site on each chromosome. The 10-Mb window size was used to calculate the recombination rate. The correlation coefficient for recombination rates in the two populations was r = 0.86.

RR is an important genetic character affecting breeding efficiency and is defined as the ratio of genetic and physical distances in the whole genome of populations. The RR varied among chromosomal segments, and significantly different RR values were observed in the RILs of both populations. In B73/By804 populations, the average minimum and maximum rates of two adjacent markers on different chromosomes were 0.08–0.11 cM/Mb and 10.43–11.50 cM/Mb, respectively. Thus, nearly 100-fold difference in RR was observed between different chromosomal regions, with varying degree of RR on different chromosomal arms. The rate of recombination was low in the centromeric regions, whereas it differed significantly on the long and short chromosome arms with increasing distance from the centromere regions. High RR values were observed in the two RILs (Figure 2). The cM/Mb values were very low in large regions around the centromeres, indicating very low recombination at these positions. The two populations were positively correlated (r = 0.86) on a whole-genome basis in terms of the average distance on chromosomes in a 10 Mb interval, but individual differences were observed at specific regions on different chromosomes (Table S5, Figure S2).

### Recombinant blocks

**Figure 3:**
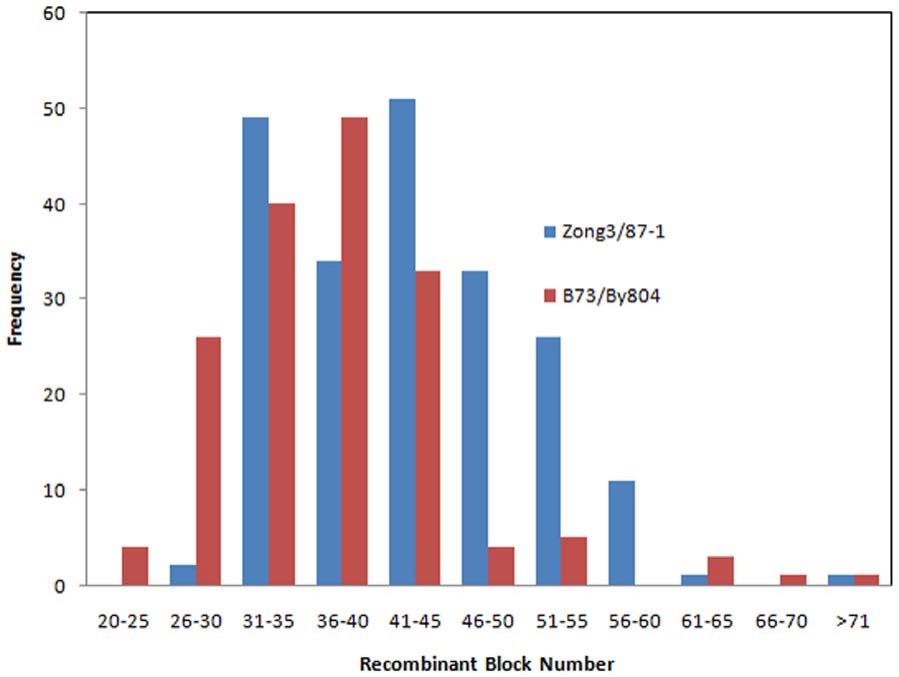
Frequency distribution of recombinant block number in the two RIL populations. Block numbers were significantly different within populations (t-test, P<0.01) and between the two populations block numbers (K-S test, P<0.01).

In theory, 3–5 recombination events on each chromosome occur in each line derived from two parents via continuous selfing [25]. On average, B73/By804 and Zong3/87-1 populations had 2.7 and 3.4 recombination events in average, respectively, on each chromosome. The number of RB was one greater than the number of recombination events on each chromosome. B73/By804 had a total of 37 RBs (from 3±1 to 5±2 RBs for each chromosome) and Zong3/87-1 had 44 RBs (from 3±1 to 6±2 RBs for each chromosome; Table S6). Physical length correlated significantly with the number of RB on each chromosome (r = 0.94 in Zong3/87-1, r = 0.87 in B73/By804), illustrating the importance of chromosome length. The number of RB varied substantially between the two populations (Figure 3). The minimum and maximum number of RBs occurred in lines H161 (22 RBs) and H059 (78 RBs) in the B73/By804 population, and in lines R213 (27 RBs) and R197 (71 RBs) in the Zong3/87-1 population. Different numbers of RBs were observed on different chromosomes in both populations, except for chromosomes 7, 8, and 9 (Table S6; Figure 3). In Zong3/87-1, the maximum number of RB occurred on chromosome 1 (1115) and the minimum on chromosome 9 (509). Chromosome 1 always had the maximum number of RB, but the minimum value varied at both the population and chromosome levels, with chromosome 10 having the minimum number of RB in B73/By804 (Table S6). In addition, entire chromosome segments in some lines were derived from the same parents without recombination events, such as for chromosome 2 of line H161 in the B73/By804 RIL populations (Figure S3).

### Resolution and power of QTL mapping using different marker densities

The Zong3/87-1 RILs were phenotyped for 11 agronomy- and yield-related traits (full details about these traits can be found in Ma et al. [21]). A total of 54 QTLs were mapped using a set of SNP+SSR markers (15.2±7.0 cM average CI), 48 QTLs were mapped using the SNP markers (19.2±9.7 cM average CI), and 42 QTLs were mapped with low-density SSR markers (33.4±10.1 cM average CI). The B73/By804 RIL population was phenotyped for 10 kernel-quality traits [22], [26], and QTL mapping was performed to identify the promising QTLs for all traits of interest. A total of 59 QTLs were mapped using the highest density marker set (SNP+SSR) with a 10.9±4.1 cM average CI, whereas the set of SNP markers used for linkage mapping revealed 55 QTLs (12.9±6.1 cM CI). There were insignificant differences in the resolution of these two sets of markers (Table 2). In contrast, the low-density markers (SSR) identified 50 QTLs for kernel-quality traits at a 21.5±11.1 cM CI (Tables 2, S7, S8).

**Table 2:** Comparison of mapped QTLs and QTL resolution using different density markers.

	Zong3/87-1			B73/By804		
	SNP+SSR	SNP	SSR	SNP+SSR	SNP	SSR
Map density (cM)	3.1	4.0	8.3	1.8	2.1	7.1
Confidence interval (cM)^a^	15.2±7.0	19.2±9.7	33.4±10.1	10.9±4.1	12.9±6.1	21.5±11.1
QTL number	54	48	42	59	55	50

a One-LOD (logarithm of odds) drop interval.

**Figure 4:**
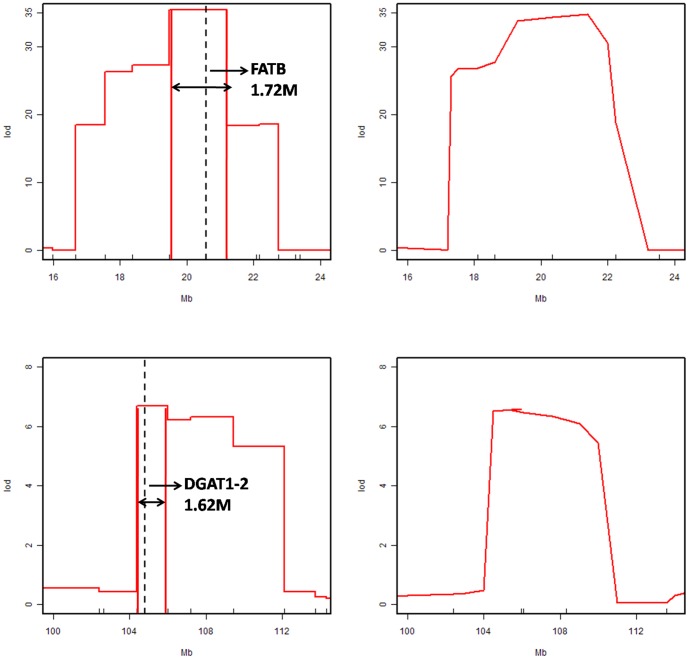
Fine mapping of two cloned genes using two different density linkage maps in the B73/By804 population. SNP+SSR markers (left graphs) and traditional SSR mapping (right graphs) used to map two different bins (top versus bottom graphs). In the two left graphs, the dotted line indicates the midpoint of two adjacent markers, and the vertical dotted line is the marker site in right graph.

Comparing the three sets of markers, the power of QTL mapping improved 28% in B73/By804 and 18% in Zong3/87-1 populations using the highest density markers (SNP+SSR). The resolution of mapping improved 54% and 49% in 1 LOD CI in the B73/By804 and Zong3/87-1 populations, respectively (Table 2). These analyses showed that higher density markers could significantly improve the power and resolution of QTL maps. More polymorphic markers were obtained within QTL CIs, which could be used for fine mapping of QTLs with a more significant effect. Two major QTLs (Pal9 and Qil6) affecting oil concentration were cloned in previous studies [28]–[29] and also segregated in B73/By804 populations. The two QTLs were used to compare QTL mapping resolution using different marker densities. QTL-Pal9 could be narrowed from 3.5 Mb to 1.72 Mb (Figure 4a, 4b) when comparing the SNP+SSR and SSR marker datasets. For QTL-Qil6, the mapping resolution increased significantly from 10.8 Mb to 1.62 Mb (Figure 4c, 4d).

## Discussion

### Application of SNPs to identify outliers

The study of genetic variation is extremely important for discovering differences in individuals within a population, particularly by using high-density markers that identify rare differences among individuals derived from the same population [30]. In such studies, the most complex and time-consuming process is the development of a population and for the purpose at hand. This multi-step process is labor-intensive, requiring careful attention to details such as when generating a RIL population. It is important to identify lines contaminated by mixed or foreign pollen during pollination. The high-density markers used in the present study substantially overcame the problem of identifying outliers and different types of pollen contamination. Identifying such lines will increase the accuracy and precision of linkage and QTL mapping.

If the outliers are not accurately identified and excluded from the analysis, this phenomenon will leads to spurious results in construction of genetic map. The marker sites can deviate within the same population, with a high number of contaminated lines and QTLs estimated in erroneous regions [31]–[32]. The highly heterogeneous lines possess higher phenotypic variation than pure RILs, and the QTL peak may deviate from the original location. Minor QTLs can be drastically affected as QTL power and resolution increase, whereas high-density markers can be used to exclude the mixed lines and precisely map a QTL through linkage mapping [33]. The results showed that extensive information could be obtained using more advanced molecular markers rather than the traditional ones. Combining both sets of markers could extensively reveal the resolution and genetic information. The linkage maps of SNP+SSR, SNP and SSR markers showed nearly 4-fold and 3-fold improved resolution, respectively; with little changes in the total genetic length.

### Recombination and its implication in maize breeding

RRs and RBs affect the genetic architecture of different germplasm and are the main sources of variation, prompting scientists to methodically investigate more advanced techniques and different density markers. Uncovering the basis of these traits can reveal genetic diversity in maize and be useful for breeding when complemented with developing sequence technology. The breeding community wants to increase the rate of recombination for effective selection and increased performance of particular traits. Increasing the number of recombination will enhance the chances of variation in the germplasm which facilitate selection of desirable germplasm for improving maize genetics. RRs and RBs measure recombination, and we have observed that these traits are controlled by particular genes [34]–[35]. These genes can be fine mapped and cloned to control the pace of recombination in our breeding material and introduce more variation that may increase the possibility of developing an ideal trait. These genes could be expressed in one line to study the consequences for improving different traits.

In our study, whole fragments were inherited without recombination from the parent in some lines, although we observed an average recombination of 3–5 on 9 chromosomes, almost similar results were observed in the nested association mapping populations [17]. This phenomenon illustrated that the number of effective recombination sites was limited in the breeding process of “second cycle line” within groups [25]. These results also showed that there is a high possibility of improving the total number of recombination in breeding programs and obtaining more and better recombined materials by expanding the populations and using high-density markers to identify.

### High-density markers used to identify gametophytic factors (GA) genes

Selective elimination of zygotes, pollen lethal effects, pollen tube competition, and preferential fertilization are some of the mechanisms which could affect the phenomenon of SDR [36]–[37]. The most frequently reported SDRs are gametophytic factors in maize [37], five of which have been identified to date (http://www.maizegdb.org/) and only the *ga1* gene has been fine mapped in maize [38]. GA genes can affect the cross incompatibility, and the pollen selective effect can lead to an abnormal segregation ratio of progeny in different populations. We observed some overlapped regions of SDRs, whereas some of the SDRs were specific to a particular population, showing the uneven distribution of GA genes in different regions of different lines. However, some of the GA genes will be common in different lines of several populations. These phenomena were also observed in 25 RIL nested association mapping populations [17].

High-density markers would be more useful to quickly identify and clone GA genes. The advantages of using high-density markers include considerable reduction in the region of each SDR and capturing the total recombination occurring on different chromosome fragments. Low-density markers cannot reduce the SDR QTL to the same length as the high-density markers and gene cloning is more difficult. The GA gene peaks of each SDR can be easily identified with high-density markers such as the SDRs on chromosome 3 in Zong3/87-1 populations.

We observed several GA genes on different chromosomes in these two RIL populations, such as *ga7* on chromosome 3 and *ga8* on chromosome 9, while one significant SDR was found on chromosome 1. This SDR was also found in B73× teosinte populations using an SSR linkage map [39]. The SDR information and cloning the GA genes can help plant breeders to solve the problem of incompatibility in different germplasms. Furthermore, these genes can perform a functional role in maintaining the purity of a specific germplasm, thereby avoiding contamination by foreign pollen in future breeding programs. This phenomenon can significantly improve the process of developing germplasm for genetic investigations.

### High-density markers can improve QTL mapping power and resolution

The QTL CI of primary populations is about 10–30 cM with low-density markers [18], [40]–[41], and fine mapping is an exhaustive and cumbersome task, especially in maize. For major QTLs, fine mapping with high-resolution markers can now be done in primary populations. Barcode sequencing yields high-density SNP markers in rice RILs, and the chromosome fragments occur in different recombination bins [19]. Using this approach, QTLs for plant height and yield have been mapped to a very narrow region of about 100 kb and 200 kb, respectively [19], [42]. In the present study, two cloned QTL regions were narrowed down: QTL Pa19 (*FatB* gene controlling palmitic acid) from 3.5 Mb to 1.72 Mb, and QTL Oil6 (*DGAT1-2* gene controlling oil concentration) reduced from 10.8 Mb to 1.62 Mb. Thus, the utilization of high-density markers can dramatically increase the efficiency and resolution of QTL maps.

QTL mapping can be the best way for cloning genes if ultra-high density markers can be used to ensure the exact recombination breakpoint. Furthermore, ultra-high density markers can divide the chromosomal segments into small recombination bins, and each bin can be associated with a specific trait. Theoretically big population size and more markers will increase the resolution of QTL mapping. Large population will have more recombination and more markers will be required for identification of all the recombination and get high resolution. But for a specific population the number of markers must be fixed to divulge the total recombination with best resolution [43]. The limited recombination of segregation populations [25], [43] means that QTL mapping should be done with association mapping to obtain more valid results for gene identification and association with specific traits. In the near future, we will be able to use the bin map for association mapping after using high-density marker bin map construction. The bin will usually be up to 200 kb using the MaizeSNP50 chip [44] for construction of an ultra-high density bin map in maize. The high-density markers in segregating populations will enable identification of all recombination events and QTLs into a very small region. In association mapping populations, the chromosomal region is usually very small with a low QTL detection power. Hence, these two approaches should be combined to avoid the cumbersome task of determining population structure and obtaining false positive results. Joint linkage and association mapping have been successfully used [28], and QTLs can be identified with high resolution.

## Supporting Information

Figure S1 **Lines Non-B73 and Non-By804.** H065 is a line that is a pure progeny of B73/By804, and we calculate the chromosome fragments rate derived from B73, By804, and unknown lines.
(TIF)

Figure S2 **Correlation of recombination rate in a 10-Mb window size of two populations.** The *x* axis and *y* axis show cM/Mb values for a 10-Mb region in the B73/By804 and Zong3/87-1 populations, respectively.
(TIF)

Figure S3 **Number of recombination events in two different RILs.** For lines H161, H163, and H121, the red and blue segments were derived from B73 and By804, respectively. For lines R213, R234, and R165, the red and blue segments were derived from Zong3 and 87-1, respectively. The black dot delegated the other lines in two populations.
(TIF)

Table S1 **Linkage map with different density markers in the B73/By804 populations.**
(DOCX)

Table S2 **Linkage map with different density markers in the Zong3/87-1 populations.**
(DOCX)

Table S3 **Total number of segregation distortion markers in the two RIL populations.** ^a^Number of SDL. ^b^Number of markers in the linkage map.
(DOCX)

Table S4 **SDRs identified in two RIL populations.** ^a^SDR interval. ^b^ Parent from which each SDR derived.
(DOCX)

Table S5 **Recombination frequency distribution in the whole genome of the two populations.**
(DOCX)

Table S6 **Recombinant block number variance in the two RIL populations.** ^a^Number of all lines in the populations. ^b^Number of recombinant blocks in each chromosome.
(DOCX)

Table S7 **QTLs identified for 10 kernel quality traits from the RIL population of the B73/By804 cross using the SNP+SSR, SNP, and SSR maps, with a 2.5 LOD (logarithm of odds) threshold.**
(XLS)

Table S8 **QTLs identified for 11 agronomy and yield traits from the RIL population of the Zong3/87-1 cross using the SNP+SSR, SNP, and SSR maps, with a 2.5 LOD (logarithm of odds) threshold.**
(XLS)
