# Supplementary figures and images for: Exploring the Genetic Characteristics of Two Recombinant Inbred Line Populations via High-Density SNP Markers in Maize
Source: PLoS One. 2012 Dec 27;7(12):e52777. doi: 10.1371/journal.pone.0052777 (PMC3531342; doi:10.1371/journal.pone.0052777)

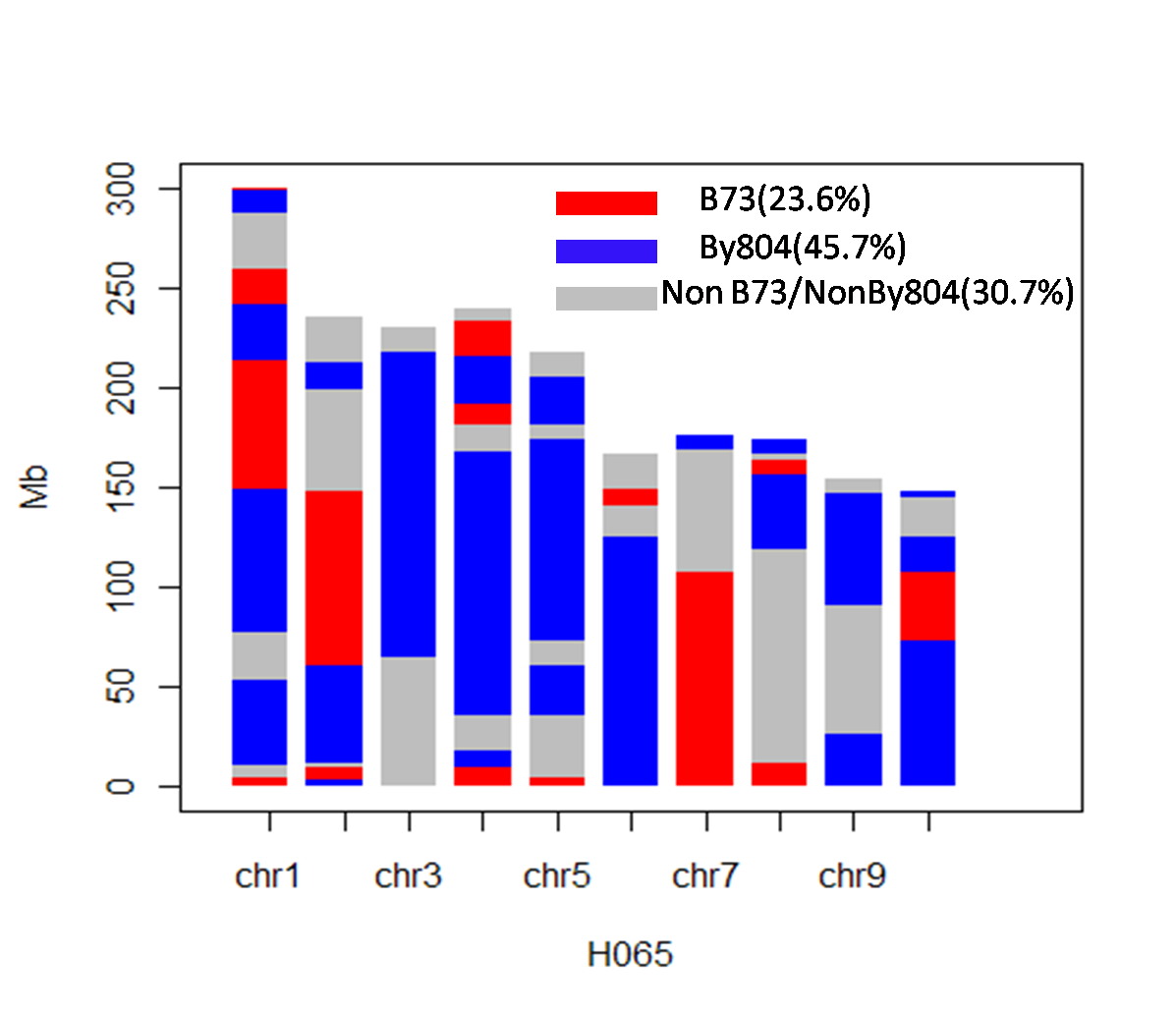

Supplement: Figure S1 — Lines Non-B73 and Non-By804. H065 is a line that is a pure progeny of B73/By804, and we calculate the chromosome fragments rate derived from B73, By804, and unknown lines. (TIF) [file pone.0052777.s001.tif]

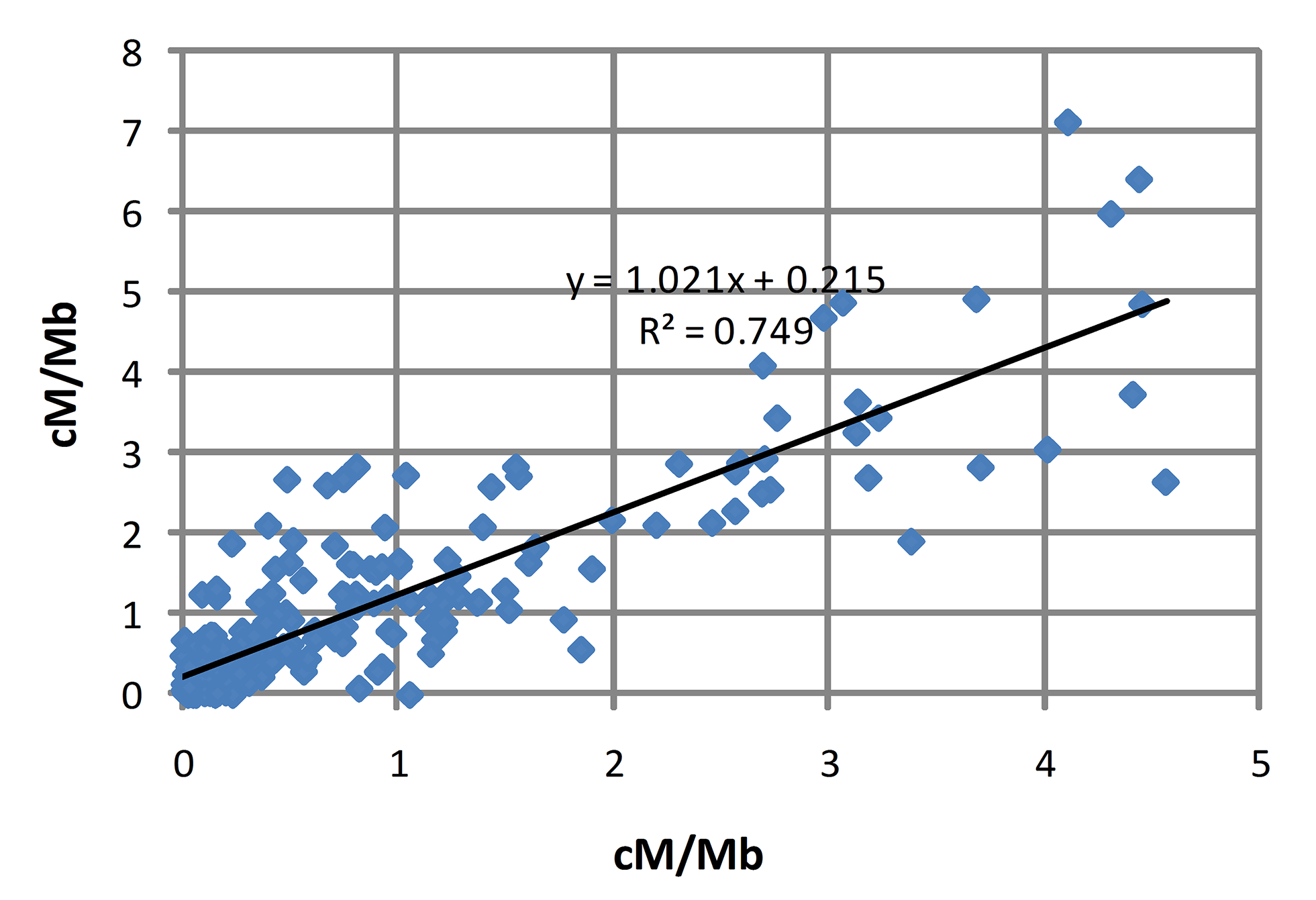

Supplement: Figure S2 — Correlation of recombination rate in a 10-Mb window size of two populations. The x axis and y axis show cM/Mb values for a 10-Mb region in the B73/By804 and Zong3/87-1 populations, respectively. (TIF) [file pone.0052777.s002.tif]

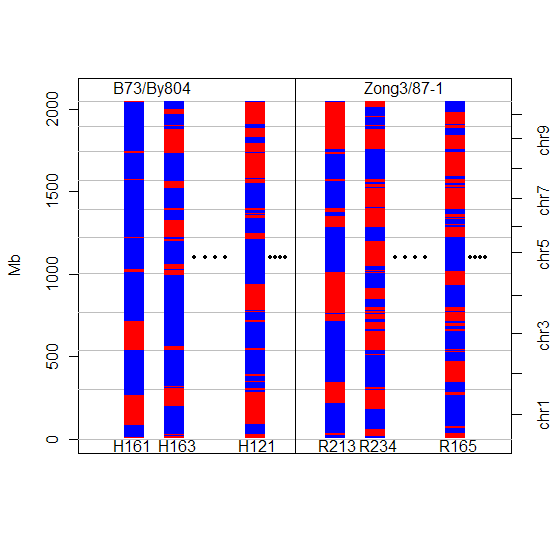

Supplement: Figure S3 — Number of recombination events in two different RILs. For lines H161, H163, and H121, the red and blue segments were derived from B73 and By804, respectively. For lines R213, R234, and R165, the red and blue segments were derived from Zong3 and 87-1, respectively. The black dot delegated the other lines in two populations. (TIF) [file pone.0052777.s003.tif]
