# Supplementary material for: Exploring the Genetic Characteristics of Two Recombinant Inbred Line Populations via High-Density SNP Markers in Maize
Source: PLoS One. 2012 Dec 27;7(12):e52777. doi: 10.1371/journal.pone.0052777 (PMC3531342; doi:10.1371/journal.pone.0052777)
Supplement: Table S1 — Linkage map with different density markers in the B73/By804 populations. (DOCX) [file pone.0052777.s004.docx]

**Table S1.** Linkage map with different density markers in the B73/By804 populations.

| Chromosome | Markers  SNP + SSR (SNP) (SSR) | Length (cM)  SNP + SSR (SNP) (SSR) | Average length (cM)  SNP + SSR (SNP) (SSR) |
| --- | --- | --- | --- |
| chr1 | 139 (112) (27) | 218.2 (204.4) (209.4) | 1.6 (1.8) (8.1) |
| chr2 | 94 (74) (20) | 174.1 (154.7) (159.2) | 1.9 (2.1) (8.4) |
| chr3 | 79 (63) (16) | 174.0 (150.6) (125.3) | 2.2 (2.4) (8.4) |
| chr4 | 85 (70) (15) | 148.4 (136.0) (122.0) | 1.8 (2.0) (8.7) |
| chr5 | 94 (78) (16) | 147.9 (146.3) (143.5) | 1.6 (1.9) (9.6) |
| chr6 | 70 (45) (25) | 137.9 (88.5) (142.5) | 2.0 (2.0) (5.9) |
| chr7 | 62 (48) (14) | 126.0 (102.9) (111.5) | 2.1 (2.2) (8.6) |
| chr8 | 89 (65) (24) | 166.7 (158.7) (147.4) | 1.9 (2.5) (6.4) |
| chr9 | 78 (52) (26) | 131.7 (117.4) (87.1) | 1.7 (2.3) (3.5) |
| chr10 | 61 (46) (15) | 101.7 (95.2) (88.4) | 1.7 (2.1) (6.3) |
| Overall | 851 (653) (198) | 1526.7 (1354.7) (1336.3) | 1.8 (2.1) (7.1) |
