# Supplementary material for: Exploring the Genetic Characteristics of Two Recombinant Inbred Line Populations via High-Density SNP Markers in Maize
Source: PLoS One. 2012 Dec 27;7(12):e52777. doi: 10.1371/journal.pone.0052777 (PMC3531342; doi:10.1371/journal.pone.0052777)
Supplement: Table S2 — Linkage map with different density markers in the Zong3/87-1 populations. (DOCX) [file pone.0052777.s005.docx]

**Table S2.** Linkage map with different density markers in the Zong3/87-1 populations.

| Chromosome | Markers  SNP + SSR (SNP) (SSR) | Length (cM)  SNP + SSR (SNP) (SSR) | Average length (cM)  SNP + SSR (SNP) (SSR) |
| --- | --- | --- | --- |
| chr1 | 92 (62) (30) | 316.0 (264.6) (273.6) | 3.5 (4.3) (9.4) |
| chr2 | 63 (44) (19) | 217.4 (169.2) (156.3) | 3.5 (3.9) (8.7) |
| chr3 | 77 (45) (32) | 232.7 (200.6) (217.9) | 3.1 (4.6) (7.0) |
| chr4 | 65 (41) (24) | 231.7 (191.7) (206.7) | 3.6 (4.8) (9.0) |
| chr5 | 98 (75) (23) | 228.4 (219.5) (209.0) | 2.4 (3.0) (9.5) |
| chr6 | 45 (31) (14) | 148.1 (130.0) (113.7) | 3.4 (4.3) (8.7) |
| chr7 | 47 (29) (18) | 179.2 (139.2) (151.4) | 3.9 (5.0) (8.9) |
| chr8 | 72 (53) (19) | 168.4 (150.2) (110.8) | 2.4 (2.9) (6.1) |
| chr9 | 46 (37) (9) | 110.3 (111.0) (50.6) | 2.5 (3.1) (6.3) |
| chr10 | 44 (22) (22) | 164.1 (152.9) (171.3) | 3.8 (7.3) (8.2) |
| Overall | 649 (439) (210) | 1996.2 (1728.8) (1661.2) | 3.1 (4.0) (8.3) |
