# Supplementary material for: Exploring the Genetic Characteristics of Two Recombinant Inbred Line Populations via High-Density SNP Markers in Maize
Source: PLoS One. 2012 Dec 27;7(12):e52777. doi: 10.1371/journal.pone.0052777 (PMC3531342; doi:10.1371/journal.pone.0052777)
Supplement: Table S3 — Total number of segregation distortion markers in the two RIL populations. aNumber of SDL. bNumber of markers in the linkage map. (DOCX) [file pone.0052777.s006.docx]

**Table S3.** Total number of segregation distortion markers in the two RIL populations.

|  | B73/By804 | |  | Zong3/87-1 | |
| --- | --- | --- | --- | --- | --- |
| Chromosome | Deviated markers^a^ | No. markers^b^ |  | Deviated markers | No. markers |
| chr1 | 43 | 139 |  | 59 | 92 |
| chr2 | 29 | 94 |  | 19 | 63 |
| chr3 | 24 | 79 |  | 10 | 77 |
| chr4 | 9 | 85 |  | 2 | 65 |
| chr5 | 1 | 94 |  | 4 | 98 |
| chr6 | 28 | 70 |  | 1 | 45 |
| chr7 | 1 | 62 |  | 9 | 47 |
| chr8 | 37 | 89 |  | 2 | 72 |
| chr9 | 64 | 78 |  | 12 | 46 |
| chr10 | 8 | 61 |  | 4 | 44 |
| Overall | 244 | 851 |  | 122 | 649 |

^a^Number of SDL.

^b^Number of markers in the linkage map.
