# Supplementary material for: Exploring the Genetic Characteristics of Two Recombinant Inbred Line Populations via High-Density SNP Markers in Maize
Source: PLoS One. 2012 Dec 27;7(12):e52777. doi: 10.1371/journal.pone.0052777 (PMC3531342; doi:10.1371/journal.pone.0052777)
Supplement: Table S4 — SDRs identified in two RIL populations. aSDR interval. b Parent from which each SDR derived. (DOCX) [file pone.0052777.s007.docx]

**Table S4**. SDRs identified in two RIL populations.

| Number | Chromosome | Location (Mb)^a^ | Parent^b^ |
| --- | --- | --- | --- |
| 1 | chr1 | 161.0–228.2 | B73 |
| 2 | chr1 | 73.9–148.6 | B73 |
| 3 | chr2 | 169.5–173.8 | By804 |
| 4 | chr2 | 110.8–147.3 | By804 |
| 5 | chr2 | 20.1–29.7 | By804 |
| 6 | chr2 | 6.0–15.6 | By804 |
| 7 | chr3 | 178.2–214.3 | B73 |
| 8 | chr4 | 162.1–178.9 | By804 |
| 9 | chr6 | 113.6–125.5 | B73 |
| 10 | chr6 | 73.9–85.8 | By804 |
| 11 | chr6 | 33.0–57.5 | By804 |
| 12 | chr8 | 6.7–12.6 | B73 |
| 13 | chr8 | 24.0–100.9 | B73 |
| 14 | chr8 | 115.1–130.5 | B73 |
| 15 | chr9 | 146.7–146.9 | By804 |
| 16 | chr9 | 12.3–145.3 | By804 |
| 17 | chr9 | 11.5–11.6 | By804 |
| 18 | chr10 | 85.2–99.6 | B73 |
| 19 | chr1 | 292.5–296.0 | 87-1 |
| 20 | chr1 | 83.7–258.3 | 87-1 |
| 21 | chr1 | 6.2–22.5 | Zong3 |
| 22 | chr2 | 215.1–229.5 | 87-1 |
| 23 | chr2 | 185.3–200.1 | 87-1 |
| 24 | chr2 | 2.4–3.1 | Zong3 |
| 25 | chr3 | 1.3–7.4 | Zong3 |
| 26 | chr7 | 135.5–148.8 | Zong3 |
| 27 | chr9 | 28.6–93.8 | 87-1 |
| 28 | chr10 | 4.9–5.5 | Zong3 |

^a^SDR interval.

^b^ Parent from which each SDR derived.
