# Supplementary material for: Exploring the Genetic Characteristics of Two Recombinant Inbred Line Populations via High-Density SNP Markers in Maize
Source: PLoS One. 2012 Dec 27;7(12):e52777. doi: 10.1371/journal.pone.0052777 (PMC3531342; doi:10.1371/journal.pone.0052777)
Supplement: Table S5 — Recombination frequency distribution in the whole genome of the two populations. (DOCX) [file pone.0052777.s008.docx]

**Table S5**. Recombination frequency distribution in the whole genome of the two populations.

| Chromosome |  | B73/By804 | |  | Zong3/87-1 | |
| --- | --- | --- | --- | --- | --- | --- |
|  | Min  cM/Mb | Max  cM/Mb | Mean  cM/Mb | Min  cM/Mb | Max  cM/Mb | Mean  cM/Mb |
| chr1 | 0.03 | 8.57 | 1.31 | 0.01 | 10.86 | 1.89 |
| chr2 | 0.12 | 14.59 | 1.98 | 0.02 | 17.78 | 2.16 |
| chr3 | 0.64 | 7.41 | 1.39 | 0.15 | 8.80 | 1.92 |
| chr4 | 0.08 | 9.92 | 1.54 | 0.06 | 10.81 | 2.18 |
| chr5 | 0.01 | 12.03 | 1.94 | 0.04 | 8.76 | 2.16 |
| chr6 | 0.01 | 16.90 | 1.65 | 0.16 | 15.05 | 2.28 |
| chr7 | 0.01 | 7.72 | 1.93 | 0.05 | 9.79 | 2.41 |
| chr8 | 0.05 | 10.44 | 2.38 | 0.08 | 10.09 | 2.13 |
| chr9 | 0.02 | 9.85 | 2.10 | 0.08 | 6.79 | 1.50 |
| chr10 | 0.03 | 6.84 | 1.83 | 0.12 | 16.71 | 2.81 |
| Mean | 0.10 | 10.43 | 1.81 | 0.08 | 11.50 | 2.14 |
