# Supplementary material for: Exploring the Genetic Characteristics of Two Recombinant Inbred Line Populations via High-Density SNP Markers in Maize
Source: PLoS One. 2012 Dec 27;7(12):e52777. doi: 10.1371/journal.pone.0052777 (PMC3531342; doi:10.1371/journal.pone.0052777)
Supplement: Table S6 — Recombinant block number variance in the two RIL populations. aNumber of all lines in the populations. bNumber of recombinant blocks in each chromosome. (DOCX) [file pone.0052777.s009.docx]

**Table S6.** Recombinant block number variance in the two RIL populations.

| Chromosome | Zong3/87-1 (n^a^ = 174) | | B73/By804 (n = 166) | |
| --- | --- | --- | --- | --- |
|  | Block No.^b^ | Mean ± s.d. | Block No. | Mean ± s.d. |
| chr1 | 1115 | 6 ± 2 | 838 | 5 ± 2 |
| chr2 | 814 | 5 ± 2 | 688 | 4 ± 2 |
| chr3 | 890 | 5 ± 2 | 663 | 4 ± 2 |
| chr4 | 871 | 5 ± 2 | 615 | 4 ± 2 |
| chr5 | 881 | 5 ± 2 | 614 | 4 ± 2 |
| chr6 | 623 | 4 ± 2 | 566 | 3 ± 2 |
| chr7 | 692 | 4 ± 2 | 535 | 4 ± 2 |
| chr8 | 693 | 4 ± 2 | 670 | 4 ± 2 |
| chr9 | 509 | 3 ± 1 | 564 | 3 ± 2 |
| chr10 | 673 | 4 ± 2 | 473 | 3 ± 1 |
| Total | 7761 | 44 ± 7 | 6226 | 37 ±7 |

^a^Number of all lines in the populations.

^b^Number of recombinant blocks in each chromosome.
